# An Efficient Chemical Synthesis of Scutellarein: An *in Vivo* Metabolite of Scutellarin

**DOI:** 10.3390/molecules21030263

**Published:** 2016-02-25

**Authors:** Ze-Xi Dong, Nian-Guang Li, Peng-Xuan Zhang, Ting Gu, Wen-Yu Wu, Zhi-Hao Shi

**Affiliations:** 1 Jiangsu Collaborative Innovation Center of Chinese Medicinal Resources Industrialization, Jiangsu Key Laboratory for High Technology Research of TCM Formulae, National and Local Collaborative Engineering Center of Chinese Medicinal Resources Industrialization and Formulae Innovative Medicine, Nanjing University of Chinese Medicine, Nanjing 210023, Jiangsu, China; dongzexi1215@163.com (Z.-X.D.); zpx20130901@126.com (P.-X.Z.); guting1992@163.com (T.G.); 15150513147@163.com (W.-Y.W.); 2 Department of Organic Chemistry, China Pharmaceutical University, Nanjing 211198, Jiangsu, China

**Keywords:** scutellarin, scutellarein, metabolite, efficient, synthesis

## Abstract

Scutellarein (**2**), which is an important *in vivo* metabolite of scutellarin (**1**), was synthesized from 3,4,5-trimethoxyphenol (**3**) in high yield in four steps. This strategy relies on acetylation, aldolization, cyclization and hydrolysis reactions, respectively.

## 1. Introduction

**Figure 1:**
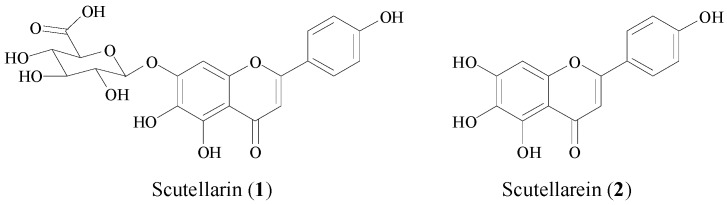
Chemical structures of scutellarin (**1**) and scutellarein (**2**).

As a frequently-occurring disease, ischemic cerebrovascular is a serious threat to human health and it has been one of the leading causes of death and disability around the world [1]. Traditional Chinese medicines are rich sources for drug lead compound discovery as they have been used clinically for thousands years. Scutellarin (**1**, Figure 1), a main active ingredient extracted from *Erigeron breviscapus* (Vant.) Hand-Mazz., which mainly grows in Yunnan Province of China, has been wildly used to treat acute cerebral infarction and paralysis induced by cerebrovascular diseases such as hypertension, cerebral thrombosis and cerebral hemorrhage in China since 1984 [2]. Interestingly, scutellarin (**1**) is mainly hydrolyzed into scutellarein (**2**, Figure 1) in the intestine [3], and scutellarein (**2**) was much more easily absorbed than scutellarin (**1**) after oral administration of both of them in equal doses [4]. In previous studies, our research group has found that scutellarein (**2**) had better protective effects than scutellarin (**1**) against neuronal injury in a rat cerebral ischemia model [5,6].

Unfortunately, scutellarein (**2**) is not readily available commercially, so the chemical synthesis of this metabolite has become important in recent years. We have previously synthesized scutellarein (**2**) from scutellarin (**1**) by hydrolysis with 6N HCl in 90% ethanol under reflux, however, the yield was very low (only 17%) [7]. Cui *et al.* [8] completed one synthetic route to scutellarein (**2**) from 2-hydroxy-4,5,6-trimethoxyacetophenone (**4**) and 4-methoxybenzoyl chloride in three steps, unfortunately, there was a by-product in this acetylation and the yield was low. Yen *et al.* [9] synthesized scutellarein (**2**) in 47% yield in five steps starting from 3,4,5-trimethoxyphenol (**3**) and acetic anhydride, then the obtained 3,4,5-trimethoxyphenol acetate was transformed into 1-(6-hydroxy-2,3,4-trimethoxyphenyl)ethanone (**4**) after Fries rearrangement.

In this paper, we report an efficient chemical synthesis of scutellarein (**2**) from 3,4,5-trimethoxyphenol (**3**) and acetic acid in only four steps and with high total yield (58%).

## 2. Results and Discussion

**Scheme 1:**
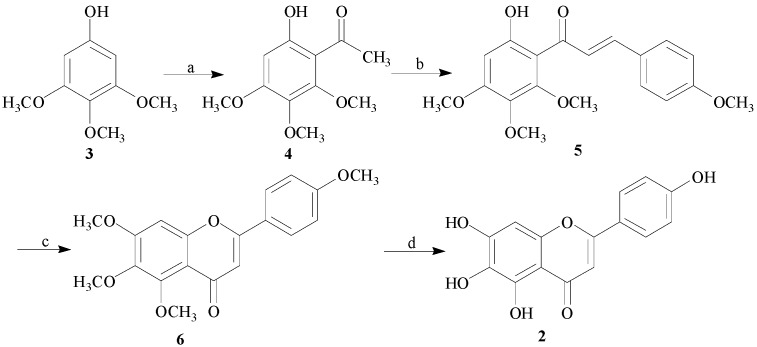
The efficient chemical synthesis of scutellarein (**2**).

As shown in Scheme 1, the starting material 3,4,5-trimethoxyphenol (**3**, 200 mg, 1.09 mmol) was first reacted with acetic acid (1 mL) in boron trifluoride diethyl etherate (5 mL) under a N_2_ atmosphere at 85 °C, to afford a high yield (92%) of the desired Friedel Crafts acylation reaction product **4**. Next, compound **5** was synthesized by a base-catalyzed Claisen-Schmidt condensation reaction of **4** and 4-methoxybenzaldehyde. Fortunately, the cyclization of **5** produced the desired compound **6** in 82% yield, using iodine as the catalyst in dimethyl sulfoxide solution at 100 °C for 2 h. Finally, the demethylation of **6** with 40% HBr in the refluxing CH_3_COOH led to scutellarein (**2**) in 90% yield, for a total overall yield of 58%.

## 3. Experimental Section

### 3.1. General Information

Reagents and solvents were purchased from commercial sources and used without further purification unless otherwise specified. Air- and moisture-sensitive liquids and solutions were transferred via syringe or stainless steel cannula. Organic solutions were concentrated by rotary evaporation (BuChi R-3, Surat, India) below 45 °C at approximately 20 mm Hg. All non-aqueous reactions were carried out under anhydrous conditions using flame-dried glassware in an argon atmosphere in dry, freshly distilled solvents, unless otherwise noted. Yields refer to chromatographically and spectroscopically (^1^H-NMR) homogeneous materials, unless otherwise stated. Reactions were monitored by thin-layer chromatography (TLC) carried out on 0.15–0.20 mm silica gel plates (RSGF 254, Yantai, China) using UV light as the visualizing agent. The melting points (m.p.) were measured on a WRS-1B apparatus (Hangzhou, China) and are not corrected. ^1^H-NMR (300 MHz) and ^1^^3^C-NMR spectra (75 MHz) were obtained with a Bruker AV-300 spectrometer (Karlsruhe, Germany). Chemical shifts are recorded in ppm downfield from tetramethylsilane. *J* values are given in Hz. Abbreviations used are s (singlet), d (doublet), t (triplet), q (quartet), b (broad) and m (multiplet).

### 3.2. Synthesis

*1-(6-Hydroxy-2,3,4-trimethoxyphenyl)ethanone* (**4**): To a stirring solution of **3** (10 g, 54.5 mmol) in BF_3_·Et_2_O (25 mL) was added CH_3_COOH (15 mL). After stirring under N_2_ atmosphere at 85 °C for 2.5 h, the reaction mixture was allowed to warm to room temperature. Then ice water (50 mL) was added slowly. The mixture was extracted with ethyl acetate (30 mL) three times, and the organic layer was dried over Na_2_SO_4_, filtered and concentrated to afford **4** (11.27 g, 92% yield) as a yellow solid [9]. M.p. 147–148 °C. ^1^H-NMR (CDCl_3_) δ 6.28 (s, 1H, 5-H), 4.10 (s, 3H, 2-H), 3.97 (s, 3H, 3-H), 3.77 (s, 3H, 4-H), 2.81 (s, 3H, 2′-H); ^13^C-NMR (CDCl_3_) δ 203.5 (CO), 161.9 (C(4)), 160.7 (C(6)), 155.4 (C(2)), 134.5 (C(3)), 108.1 (C(1)), 96.1 (C(5)), 61.0 (MeO-C(2,2′)), 56.4 (MeO-C(3)), 31.4 (MeO-C(4)); ESI-MS: *m*/*z* 227 [M + H]^+^.

*1-(6-Hydroxy-2,3,4-trimethoxyphenyl)-3-(4-methoxyphenyl)prop-2-en-1-one* (**5**): 4-Methoxybenzaldehyde (8 mL, 66 mmol, 1.5 equiv) was added to a stirring mixture of **4** (10 g, 44 mmol) and *t*-BuOK (12.32 g, 110 mmol, 2.5 equiv) in EtOH (150 mL). The reaction mixture was refluxed gently for 4 h at 85 °C. After being cooled down to room temperature, the reaction mixture was poured into glacial water and the pH was adjusted to 3~4. Then the mixture was filtered and dried to afford **5** (13.02 g, 86% yield) as a yellow solid [9]. M.p. 135–136 °C. ^1^H-NMR (CDCl_3_) δ 13.78 (s, 1H, 6′-OH), 7.84 (s, 2H, 2,3-H), 7.59 (d, *J* = 8.7 Hz, 2H, 2′′,6′′-H), 6.92 (d, *J* = 8.7 Hz, 2H, 3′′,5′′-H), 6.29 (s, 1H, 5′-H), 3.92 (s, 3H, 2′-H), 3.90 (s, 3H, 3′-H), 3.86 (s, 3H, 4′-H), 3.83 (s, 3H, 4′′-H); ^13^C-NMR (CDCl_3_) δ 192.3 (CO), 162.1 (C(4)), 161.0 (C(6)), 160.0 (C(4′)), 155.4 (C(2)), 143.7 (CO-C=*C*H), 135.5 (C(3)), 130.6 (C(2′,6′)), 127.9 (C(1′)), 124.2 (CO-*C*=CH), 114.8 (C(3′,5′)), 108.3 (C(1)), 96.1 (C(5)), 62.0 (MeO-C(2)), 61.4 (MeO-C(3)), 56.4 (MeO-C(4)), 55.1 (MeO-C(4′)); ESI-MS: *m*/*z* 345 [M + H]^+^.

*5,6,7-Trimethoxy-2-(4-methoxyphenyl)-4H-chromen-4-one* (**6**): To a solution of **5** (10 g, 29 mmol) dissolved in DMSO (40 mL) was added I_2_ (0.74 g) with vigorous stirring at 100 °C. After 2 h, the reaction mixture was poured into crashed ice water (30 mL). The precipitate was filtered and washed by 10% aq. Na_2_S_2_O_3_ soln, followed by recrystallization in EtOH to afford **6** (8.13 g, 82% yield) as a yellow solid [9]. M.p. 140–141 °C. ^1^H-NMR (CDCl_3_) δ 7.82 (d, *J* = 8.9 Hz, 2H, 2′,6′-H), 7.00 (d, *J* = 8.9 Hz, 2H, 3′,5′-H), 6.80 (s, 1H, 3-H), 6.61 (s, 1H, 8-H), 3.99 (s, 3H, 5-H), 3.98 (s, 3H, 6-H), 3.92 (s, 3H, 7-H), 3.89 (s, 3H, 4′-H); ^13^C-NMR (CDCl_3_) δ 177.3 (C(4)), 162.4 (C(2)), 161.0 (C(4′)), 157.7 (C(7)), 154.4 (C(8a)), 152.3 (C(5)), 140.1 (C(6)), 127.7 (C(2′,6′)), 123.7 (C(1′)), 114.3 (C(3′,5′)), 112.8 (C(4a)), 106.9 (C(3)), 96.3 (C(8)), 62.1 (MeO-C(5)), 61.6 (MeO-C(6)), 56.2 (MeO-C(7)), 55.4 (MeO-C(4′)); ESI-MS: *m*/*z* 343 [M + H]^+^.

*4',5,6,7-Tetrahydroxyflavone* (**2**): To a solution of **6** (8 g, 23.2 mmol) dissolved in CH_3_COOH (100 mL) was added 40% HBr (50 mL) with stirring at 120 °C for 24 h. After cooled down to the room temperature, the reaction mixture was poured into ice water (50 mL). The precipitate was filtered and washed by water and then recrystallized by EtOH to afford **2** (5.97 g, 90% yield) as a yellow solid [9]. M.p. 160–141 °C. ^1^H-NMR (DMSO-*d*_6_) δ 12.79 (s, 1H, 5-OH), 10.44 (s, 1H, 7-OH), 10.30 (s, 1H, 4′-OH), 8.71 (s, 1H, 6-OH), 7.90–7.93 (d, 2H, *J* = 8.8 Hz, 2′,6′-H), 6.90–6.93 (d, 2H, *J* = 8.8 Hz, 3′,5′-H), 6.78 (s, 1H, 3-H), 6.73 (s, 1H, 8-H); ^13^C-NMR (DMSO-*d*_6_) δ 182.3 (C(4)), 162.4 (C(2)), 161.0 (C(4′)), 153.7 (C(7)), 149.4 (C(8a)), 147.3 (C(5)), 129.1 (C(6)), 128.7 (C(2′,6′)), 121.7 (C(1′)), 116.3 (C(3′,5′)), 104.8 (C(4a)), 102.9 (C(3)), 93.3 (C(8)); ESI-MS: *m*/*z* 287 [M + H]^+^.

## 4. Conclusions

In summary, we have developed an efficient chemical synthesis of scutellarein (**2**) in high yield in only four steps. This strategy relies on acetylation, aldolization, cyclization and hydrolysis step, respectively. This synthetic method is effective and it has industrial application value.

## Figures and Tables

*Reagents and Conditions*: (**a**) CH_3_COOH, BF_3_·Et_2_O, N_2_, reflux, 85 °C, 2.5 h, 92%; (**b**) 4-methoxybenzaldehyde (1.5 equiv), *t*-BuOK (2.5 equiv), EtOH, reflux, 85 °C, 4 h, 86%; (**c**) DMSO, I_2_, reflux, 100 °C, 2 h, 82%; (**d**) 40% hydrobromic acid, CH_3_COOH, N_2_, reflux, heat, 24 h, 90%.
